# BOTME Study: A National e‐Delphi Study on the Use of Botulinum Toxin for Aesthetic Treatment of Middle Easterners

**DOI:** 10.1111/jocd.70492

**Published:** 2025-10-13

**Authors:** Mohammed Al‐Haddab, Mohammed AlFada, Yasser Alqubaisy, Naif Alshahrani, Mohammed A. Alsufyani, Abdulaziz Madani, Abdullah Aleisa, Homaid Obidullah Alotaibi, Abdulmajeed Alajlan, Norah Alsubait, Fatimah J. Al Muqarrab

**Affiliations:** ^1^ Department of Dermatology College of Medicine, King Saud University Riyadh Saudi Arabia; ^2^ Department of Dermatology Prince Sultan Medical Military City Riyadh Saudi Arabia; ^3^ Department of Dermatology College of Medicine, Prince Sattam Bin Abdulaziz University Al‐Kharj Saudi Arabia; ^4^ Department of Dermatology King Faisal Hospital Makkah Saudi Arabia; ^5^ Department of Dermatology Aliman General Hospital Riyadh Saudi Arabia

**Keywords:** aesthetic, botox, botulinum toxin (BTX), cosmetic, Middle East, skin of color

## Abstract

**Background:**

The demand for botulinum toxin A (BTX‐A) cosmetic treatments is growing among individuals with skin of color, primarily due to increased cultural acceptance, social media influence, and patient awareness. However, there remains a lack of standardized, evidence‐based guidance on the use of BTX‐A in these populations.

**Objective:**

To develop consensus‐based clinical recommendations on aesthetic botulinum toxin injection practices in patients with skin of color.

**Methods:**

A modified e‐Delphi method was conducted with 10 board‐certified dermatologists specializing in cosmetic dermatology. Inclusion criteria required ≥ 5 years of experience and publications, or teaching activity in BTX‐A use. Two e‐Delphi rounds evaluated 29 evidence‐based statements. Consensus was defined as ≥ 70% agreement.

**Results:**

Consensus was achieved on 22 statements. Key recommendations included initiating BTX‐A treatments at ages 30–35, dosing 40–60 IU for the upper face, with 16–20 week intervals, using 2 mL of saline reconstitution per 100 IU, and employing 30‐gauge insulin syringes. Topical EMLA was collectively agreed upon for pain control. While several technical aspects, such as injection depth and under‐eye applications, remained inconclusive, participants decided on key practical precautions, including posttreatment activity restrictions.

**Conclusion:**

These consensus‐based recommendations can standardize, make BTX‐A injections safe and effective for skin of color patients, supporting more uniform and comparable treatment results.

## 1 Introduction and Rationale

Botulinum toxin A (BTX‐A) has been an increasingly sought‐after cosmetic measure among racial/ethnic populations [1, 2] due to cultural acceptance, social media influences, and patients' awareness of available cosmetic options that define their preference needs [3, 4]. Skin of color is a term used to describe different individuals with varying skin tones who share similar cutaneous properties and responses to dermatologic disorders, including their tendency to hyperpigment [5, 6, 7]. Nevertheless, it is traditionally used to describe Fitzpatrick skin types (IV–VI) [8]. Currently, there is still substantial heterogeneity and incomplete reporting on many aspects of the use of botulinum toxin for the skin of color populations, particularly the Middle Eastern communities, as outlined by a recent review [9]. Although evidence‐based treatment recommendations cannot be made, the opinions of botulinum toxin experts among the Saudi population, which encounters a wide range of skin tones, are valuable in ultimately achieving uniformity in skin color cosmetic outcomes.

This study aims to establish a consensus among a national panel of dermatologists specializing in cosmetic dermatology to provide consensus‐based recommendations on various aspects of aesthetic botulinum toxin injections for use by all physicians.

## 2 Methodology

This is an e‐Delphi consensus study, which involved an initial expert meeting followed by two online e‐Delphi rounds [10]. All study participants were informed that identifiable data would be collected, and they consented to its use for publication.

### 2.1 Study Population

Eligible participants should fulfill all three criteria:

1. The participant is a board‐certified dermatologist specializing in cosmetic injections.
2. The participant has at least 5 years of clinical experience using botulinum toxin.
3. The participant has two or more publications or ongoing research projects related to botulinum injection or a toxin injection trainer.
4. The participant has experience in treating patients with a different range of skin tones.

### 2.2 Outcomes

Consensus was defined as agreement by ≥ 75% of participants, based on the threshold established in a systematic review of Delphi study reporting standards [11]. However, given that the number of respondents was a multiple of 10, a consensus threshold of 70% was deemed appropriate. The agreement was measured using a 5‐point Likert scale as follows: (1) strongly disagree, (2) disagree, (3) neutral, (4) agree, and (5) strongly agree. Statements that did not reach the consensus threshold in the e‐Delphi rounds were revised accordingly.

### 2.3 One Survey Round

The developed survey examines cosmetic goals and various aspects of botulinum toxin injection techniques. The primary objective of each round is to identify and handle potential issues in preparation for the subsequent e‐Delphi rounds. Based on the responses collected, statements for the e‐Delphi process may be refined or modified accordingly.

### 2.4 E‐Delphi Rounds

Two e‐Delphi rounds were conducted online. To facilitate statement improvement and enhance open discussion, the voting process was intentionally non‐anonymous. After each round, participants received structured feedback, allowing them to reconsider and, if necessary, revise their initial responses in the following round. Participants also had the opportunity to suggest new statements or recommend modifications, which the founding committee reviewed and, if deemed appropriate, incorporated into subsequent rounds. At the end of the process, all participants were asked to review and approve the final draft.

### 2.5 Design of Statements

A total of 29 statements were developed for the consensus study by the founding committee (M. H., F. M.), drawing on their clinical expertise and evidence identified through a systematic search cited in a recent scoping review [9]. Each statement is supported by one or more relevant scientific publications, the references of which were made available to all participants. The statements were critically reviewed by two dermatologists with extensive experience in botulinum toxin treatments. Both the initial survey questions and the e‐Delphi statements were distributed through a secure online platform (Google Forms). Designed statements and their supporting evidence are provided in a Appendix S1.

### 2.6 Data Analysis

Data collection and analysis were conducted by a founding committee member (F.M.), who was not involved in the expert panel to avoid any potential bias. Quantitative data were analyzed using Microsoft Excel (Microsoft Corporation, 2021; Version 16.95.4), and frequency distributions were calculated for all response variables. Qualitative data from open‐ended responses were extracted and analyzed separately.

## 3 Results

**FIGURE 1:**
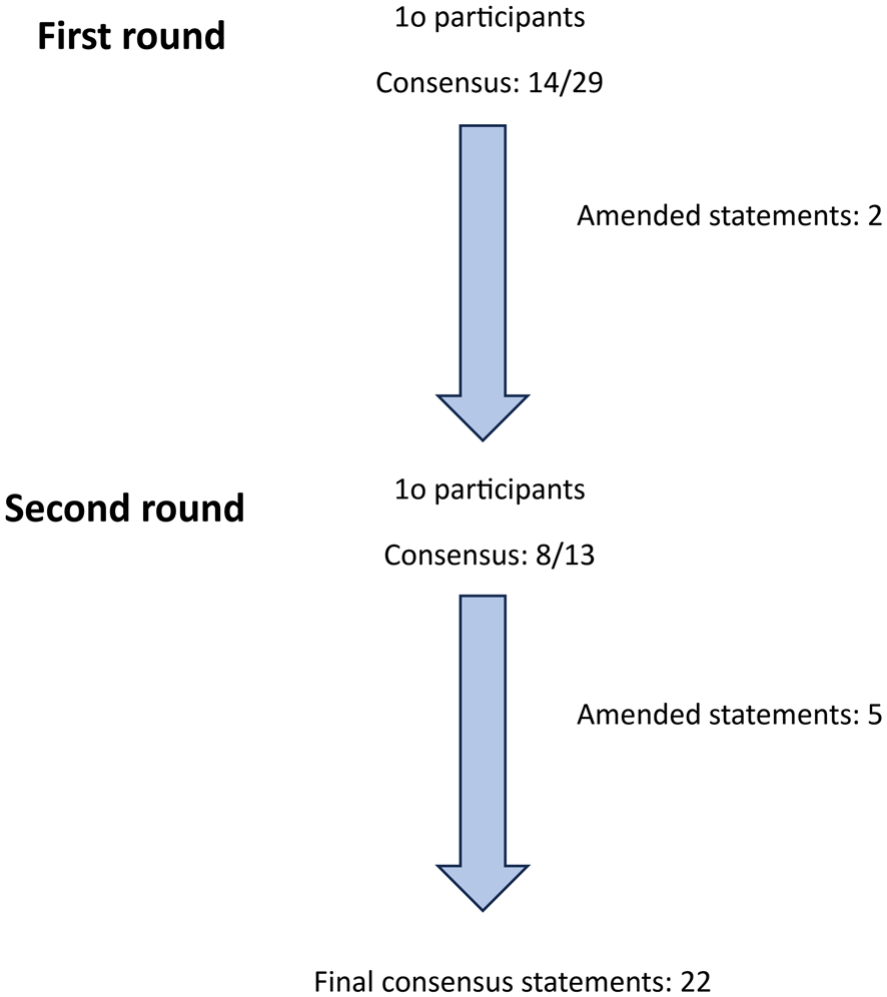
Flowchart of the e‐Delphi process including the number of rounds, the number of participants, the number of statements included, and the number of amended statements.

Invitations were sent to 19 cosmetic injection experts who met the inclusion criteria. Of these, nine did not respond, and one did not complete the first round of the e‐Delphi process. A total of 10 dermatologists completed the initial round. It was subsequently included in the complete e‐Delphi study (Figure 1). All participants completed both rounds of the e‐Delphi process, resulting in a 100% response rate. Consensus (> 70%) was achieved for 14 out of 29 statements in the first e‐Delphi round, and for an additional eight statements in the second round. The ratings from the e‐Delphi rounds, along with the voting outcomes from the committee meetings for each statement, are summarized in Table 1. Eleven statements were refined after the first e‐Delphi round, and three new statements were introduced in the second round (Figure 1).

**TABLE 1:** The final results of the e‐Delphi consensus rounds are shown with their voting results.

Statements	First	Second
Treatment goals and expectations		
*1. Facial aging, including static rhytids, appears earlier in skin types III–IV than in skin types V–VI, necessitating delaying the first botulinum toxin treatment in the latter group to the age of 30–35* [12, 13, 14, 15, 16, 17, 18]	80%	—
*2. For patients with darker skin types, facial aging concerns may focus more on changes in soft tissue volume and pigmentary alterations, rather than the development of fine and coarse wrinkles* [19]	70%	—
*3. Skin of color female patients are seeking over‐treatment of the upper face aiming at zero dynamic lines, compared to the male patients who seek minimizing the depth of the lines rather than complete elimination*	90%	—
*4. Younger female patients are usually seeking toxin injection for non‐wrinkle aesthetic indications (i.e., slimming the nostril, flipping the lips, almond eye definition, trapezius thinning)* [20]	80%	—
*5. The treatment efficacy and injection tolerance are similar between the various skin types (i.e., I–III is similar to IV–VI)* [21]	70%	—
*6. Skin type does not affect the duration of botulinum toxin efficacy. Retreatment is generally recommended every 16–20 weeks* [21, 22, 23]	50%	*90%*
*7. Aesthetic side effects of the botulinum toxin injection are more common than functional side effects*	100%	—
*8. Lower face slimming with Botox for masseteric hypertrophy is generally not a primary aesthetic concern among Middle Eastern patients* [24]	60%	*70%*
*9. Lateral eyebrow arching achieved with Botox is often considered desirable among Middle Eastern patients* [25]	50%	*100%*
*10. The strength of frown lines in Middle Eastern patients is generally comparable to that observed in other ethnic groups* [21, 25, 26, 27, 28]	60%	*90%*
11. Hookah smoking trend is increasing among young women in the region, making the perioral vertical rhytids a pronouncing cosmetic concern in the last 5 years [29]^a^	50%	—
12. The facial shape preference (i.e., oval vs. round) of Middle Eastern females are influenced by their hijab‐wearing style (i.e., hijabi vs. head cover) [24]^a^	40%	—
13. Undereye wrinkles can accentuate the appearance of dark circles, which are a significant aesthetic concern among Middle Eastern patients. The use of micro‐diluted botulinum toxin in treating fine lines may help improve their overall appearance [30, 31, 32]^a^	30%	40%
Drug and dosing		
*1. Reconstitution of the BTX using normal saline is preferred at a dilution of 2 cc of normal saline/100 IU of BTX*	100%	—
*2. On average, upper‐face Botox treatment in female patients require 40–60 units per session* [33]	30%	*90%*
*3. Switching between botulinum toxin types enhances the response to toxin when the patient starts losing efficacy*	80%	—
Equipment		
*1. Insulin syringes with 30‐gauge needles are the preferred syringes for the intra muscular injections*	80%	—
*2. Topical anesthesia in the form of EMLA cream is the preferred method of reducing the puncture pain*	100%	—
3. Topical anesthetics such as lidocaine/prilocaine (EMLA) cream should preferably be applied 2 h before BTX administration [34]^a^	10%	—
Injection techniques		
*1. Efforts should be made to minimize pain during botulinum toxin administration, as the procedure can be discomfortable* [21, 22]	70%	—
*2. Slowing the injection speed is preferable for optimum pain reduction* [35, 36]	90%	—
*3. Though patients with skin types IV–VI have a higher propensity for keloid development, I take no extra precautions compared to other patients when injecting botulinum toxin* [12, 21, 22, 33, 37, 38]	10%	*90%*
4. Occluding the supraorbital foramen with one finger is important to decrease the botulinum toxin diffusion to the levator palpebra superioris and the subsequent blepharoptosis^a^	60%	—
5. Deeper botulinum toxin injections in the frontalis muscle tend to produce better cosmetic outcomes compared to more superficial techniques^a^	40%	60%
6. Injecting the procerus muscle deeply at its bony insertion near the nasal root is preferred over targeting the muscle belly, as the latter may increase the risk of eyebrow ptosis^a^	40%	60%
7. Due to the variation in depth along the course of the corrugator supercilii, injecting too superficially medially may result in inadvertent injection of the frontalis muscle, potentially causing brow ptosis instead of elevation^a^	40%	60%
*8. Injecting the toxin in the pretarsal orbicularis oculi and the pretarsal palpebral muscles at the medial and lateral canthi to widen the eyes is a widely preferrable approach in Middle Eastern* [20]	80%	—
*9. Correcting gingival display using botulinum toxin injection is more preferable by the patients than correction of the gummy smile using soft tissue fillers. This issue can be corrected using 2.5 IU of onabotulinum toxin at each Yonsei point* [39]	80%	—
*10. The touch‐up session is usually spaced 2 weeks after the initial injection session*	60%	*90%*
11. Post botulinum toxin injection headache is experienced more frequently in skin of color patients [38]^a^	20%	—
*12. We generally advise the patients to minimize the risk of complications, avoid applying pressure, engaging in vigorous exercise, or bending during prayer (sujoud) for at least 2 h following treatment* [40, 41]	20%	*80%*
13. It is recommended to exercise the injected muscles immediately after the session to help optimize the treatment's effectiveness [42, 43, 44]^a^	—	40%

*Note:* Statements shown in italics represent items for which final consensus was reached.

^a^ Amended Statements after the e‐Delphi rounds.

Consensus was reached on the following treatment goals and expectations: delaying the first botulinum toxin treatment in the skin of color people to the age of 30–35 (80%), facial aging concerns are focused on volume replacement and pigmentary changes (70%), younger female Middle Easterners seek overtreatment of the upper face (90%), and other aesthetic goals apart from the removal of the coarse rhytides. The durability, efficacy, and side effects of botulinum toxin are comparable across different ethnic groups.

The consensus was reached that 40–60 international units (IU) of botulinum toxin are required to treat the upper face rhytides (90%), the treatment sessions have to be spaced 16–20 weeks apart (90%), the BTX reconstitution is done by diluting 100 IU of the toxin in 2 mL of normal saline (100%), and the injections are preferably done using 30 gauge insulin needles (80%). The participants agreed that switching the product type may enhance the toxin's efficacy after losing it (80%).

Moreover, consensus was reached that minimizing injection discomfort is a priority (70%), and it can be achieved using topical anesthesia (100%) and slowing the injection speed (90%). Although there was a wide variation in the injection technique of the upper face, the participants agreed on using the Yonsei point as a reference for treating gummy smiles (80%). Overall, patients are advised to avoid vigorous exercise and prolonged bending for at least 2 h after the injection session (80%).

## 4 Discussion

This study provides the first national consensus on BTX‐A use in Middle Eastern patients with skin of color. The expert panel agreed on practical recommendations regarding dosing, timing, preparation, injection techniques, and post‐procedure care. These guidelines may improve treatment consistency, safety, and patient satisfaction in the region.

A consensus was not reached regarding the use of botulinum toxin for the correction of under‐eye circles. While some experts suggested that it may help reduce the appearance of fine lines, potentially reducing the prominence of under‐eye shadows, there was insufficient agreement to support its routine use for this indication. Moreover, while immediate postinjection exercise of the muscles has been suggested in research settings as a means to enhance intramuscular uptake of the toxin, it has not been deemed beneficial or necessary in routine clinical practice [42, 43, 44]. Consensus was not reached at the location of the toxin injection. Some injectors believed that administering the injection deeply at the belly of the procerus muscle, down to the bone, may yield superior aesthetic outcomes while reducing the risk of eyebrow ptosis. However, consensus was not achieved on this technique. Similarly, in alignment with current research findings [45], no consensus was reached regarding the use of deep frontalis muscle injections. Nevertheless, 60% of respondents believed that injection depth plays a role in both enhancing the efficacy of the toxin and minimizing unintended side effects.

Consensus was reached on the importance of minimizing pain during botulinum toxin injections, with 100% agreement that topical EMLA cream is sufficient for pain management. Although topical skin cooling is a widely accepted method for reducing pain in needle‐based procedures and may offer additional benefits such as minimizing post‐injection swelling and ecchymosis [46, 47], the steering committee decided not to include it in the consensus methodology. This decision was based on the generally minimal discomfort associated with botulinum toxin injections and the theoretical concern that exposure to temperatures below 20°C could reduce the intrasynaptic translocation of botulinum toxin [48, 49]. Further well‐designed clinical studies are required to validate this hypothesis.

Despite literature‐based evidence indicating that botulinum toxin reaches the intramuscular space within 5–10 min post‐injection [40, 41], the consensus was reached on advising patients to avoid vigorous exercise, localized pressure, and prolonged bending for at least 2 h following treatment. These precautions aim to reduce the risk of unintended toxin diffusion and post‐injection bruising.

The Delphi methodology strengthened the reliability of findings despite the small panel size, as structured rounds and controlled feedback helped refine and balance expert opinions. However, consensus does not replace clinical evidence, and unresolved questions highlight the need for further studies.

## 5 Limitations

Our study has several limitations. The number of experts who completed the Delphi process was relatively small, and all participants were based in Saudi Arabia. While this gave us valuable insights into local practice, it does mean the results may not represent the full range of perspectives from other countries or regions.

Another limitation is that the process was not anonymous to the steering committee. To mitigate the influence of some opinions on others, the voting panel was held separately through individual links. In addition, only two Delphi rounds were completed. This helped us reach an agreement efficiently but may have left some of the more complex or debated issues unresolved, such as the ideal injection depth or under‐eye use of botulinum toxin.

Finally, it is important to note that our recommendations are based on expert consensus rather than clinical trial evidence. They should be seen as guidance to help standardize practice, not as definitive rules. Further research, especially studies that include patient experiences and outcomes, will be essential to strengthen and expand on the guidance we propose here.

## 6 Conclusion

This e‐Delphi consensus study offers evidence‐informed clinical recommendations on key aspects of aesthetic botulinum toxin use in patients with skin of color. These recommendations are intended for all physicians practicing cosmetology and cover treatment goals, indications, dosing, treatment intervals, syringe and needle specifications, use of local anesthesia, and manual injection techniques. Implementing these guidelines may promote greater consistency in botulinum toxin administration and improve the comparability of treatment outcomes. Furthermore, adherence to these recommendations can enhance overall treatment efficacy and patient satisfaction.

## Author Contributions

Following established authorship criteria, the authors affirm that all contributors have fully engaged in all steps of the research process and met the necessary requirements for authorship. The authors' specific distinguished contributions are those of Mohammed Alhaddab and Fatimah J. Al Muqarrab, who provided foundational insights into the study design and methodology. Yasser Alqubaisy, Mohammed A. Alsufyani, and Naif Alshahrani made significant contributions to the data analysis and interpretation of the results, while Mohammed AlFada, Abdulaziz Madani, and Abdullah Aleisa played vital roles in drafting and refining the manuscript, ensuring clarity and consistency. Homaid Obidullah Alotaibi and Abdulmajeed Alajlan provided critical feedback that enhanced the overall quality of the paper. Norah Alsubait and Fatimah J. Al Muqarrab provided essential support in coordinating the e‐Delphi rounds and assembling the final recommendations. Each author's expertise and commitment were instrumental in the successful completion of this study.

## Ethics Statement

The authors have nothing to report.

## Conflicts of Interest

The authors declare no conflicts of interest.

## Supporting information


**Appendix S1:** Supporting Information.

## Data Availability

The data that supports the findings of this study are available in the Supporting Information of this article.
