# Supplementary material for: BOTME Study: A National e‐Delphi Study on the Use of Botulinum Toxin for Aesthetic Treatment of Middle Easterners
Source: J Cosmet Dermatol. 2025 Oct 13;24(10):e70492. doi: 10.1111/jocd.70492 (PMC12516931; doi:10.1111/jocd.70492)
Supplement: Supplementary file 1 — Appendix S1: Supporting Information. [file JOCD-24-e70492-s001.docx]

First round statements

| Statements | Agreement rate |
| --- | --- |
| **Treatment Goals and expectations**   1. Facial aging, including static rhytids, appears earlier in skin types III-IV than in skin types V-VI, necessitating delaying the first botulinum toxin treatment in the latter group to the age of 30-35. | 80% |
| 1. Avoid injecting patients with skin types IV to VI, as they have a higher propensity for keloid development. | 10% |
| 1. For patients with darker skin types, facial aging concerns may focus more on changes in soft tissue volume and pigmentary alterations, rather than the development of fine and coarse wrinkles. | 70% |
| 1. Undereye circles are a major aesthetic concern for Middle Eastern patients, physicians can inject micro-diluted botulinum toxin in the fine wrinkles of the lower eyelids to minimize the under-eye circles. | 30% |
| 1. Skin of color female patients are seeking over-treatment of the upper face aiming at zero dynamic lines, compared to the male patients who seek minimizing the depth of the lines rather than complete elimination. | 90% |
| 1. Treatment of the masseteric hypertrophy is not a concern for the middle eastern patients | 60% |
| 1. The facial shape preference (i.e. oval vs round) of Middle Eastern females are influenced by their hijab-wearing style (i.e. hijabi vs head cover) | 40% |
| 1. Younger female patients are usually seeking toxin injection for non-wrinkle aesthetic indications (i.e. slimming the nostril, flipping the lips, almond eye definition, trapezius thinning). | 80% |
| 1. Inject the toxin in the pretarsal orbicularis oculi and the pretarsal palpebral muscles at the medial and lateral canthi to widen the eyes is a widely preferrable approach in Middle Eastern. | 80% |
| 1. Eyebrow arching is not desirable to Middle Eastern patients. | 50% |
| 1. Hookah smoking trend is increasing among young women in the region, making the peri-oral vertical rhytids a pronouncing cosmetic concern in the last 5 years. | 50% |
| 1. Correcting gingival display using botulinum toxin injection is more preferable by the patients than correction of the gummy smile using soft tissue fillers. This issue can be corrected using 2.5 IU of onabotulinum toxin at each Yonsei point. | 80% |
| 1. The frown lines are strong among Middle Easters, requiring an average of 15-20 IU of the toxin for their chemo denervation. | 60% |
| 1. On average, full-face treatment per session consumes 30-50 IU of the toxin. | 30% |
| 1. The touch-up session is spaced 3 weeks post the first injection session | 60% |
| 1. The botulinum toxin effect lasts longer in patients with darker skin types (IV-VI), thus, retreatment is recommended to be not more frequent than every 16-20 weeks. | 50% |
| 1. The treatment efficacy and injection tolerance are similar between the various skin types (i.e. I-III is similar to IV-VI) | 70% |
| 1. The skin of color patients experience a similar side effects profile than white patients, except in terms of headache which is experienced more frequently by the patients with skin of color. | 20% |
| 1. Aesthetic side effects of the botulinum toxin injection are more common than functional side effects. | 100% |
| 1. Minimizing pain during BTX administration should be aimed, as the treatment is often painful. | 70% |
| 1. Topical anesthesia in the form of EMLA cream is the preferable method of reducing the puncture pain. | 100% |
| 1. Topical anesthetics such as lidocaine/prilocaine (EMLA) cream should preferably be applied 2 hours before BTX administration. | 0% |
| 1. Allergan Botox is more preferable in efficacy, tolerability, and duration in skin of color patients. | 10% |
| 1. Switching between botulinum toxin types enhances the response to toxin when the patient starts losing efficacy. | 80% |
| 1. Reconstitution of the BTX using normal saline is preferred at a dilution of 2 cc of normal saline/100 IU of BTX. | 100% |
| 1. Insulin syringes with 30-gauge needles are the preferred syringes for the intra muscular injections. | 80% |
| 1. Deeper botulinum injections yield better cosmetic outcome than superficial techniques. | 40% |
| 1. Occluding the supraorbital foramen with one finger is important to decrease the botulinum toxin diffusion to the levator palpebra superioris and the subsequent blepharo-ptosis | 60% |
| 1. Slowing the injection speed is preferrable for optimum pain reduction. | 90% |
| 1. The patients are asked to minimize contact pressure (including praying) over the injected sites for the first 8 hours post injection to prevent toxin dispersion to undesirable muscles. | 20% |
